# Matrix Metalloproteinases Family Gene Polymorphisms Are Associated with Thrombosis Risk in Myeloproliferative Neoplasms

**DOI:** 10.3390/ijms26146646

**Published:** 2025-07-11

**Authors:** Roberta Vadeikienė, Aistė Savukaitytė, Danguolė Laukaitienė, Rūta Dambrauskienė, Rolandas Gerbutavičius, Elona Juozaitytė, Rasa Ugenskienė

**Affiliations:** 1 Oncology Research Laboratory, Institute of Oncology, Lithuanian University of Health Sciences, LT-50161 Kaunas, Lithuania; 2 Institute of Oncology, Lithuanian University of Health Sciences, LT-50161 Kaunas, Lithuania; 3 Department of Genetics and Molecular Medicine, Lithuanian University of Health Sciences, LT-50161 Kaunas, Lithuania

**Keywords:** myeloproliferative neoplasms, *MMP-1*, *MMP-2*, *MMP-3*, *MMP-9*, polymorphism, thrombosis

## Abstract

Myeloproliferative neoplasms (MPNs) are clonal hematopoietic disorders characterized by excessive proliferation of one or more myeloid lineages, frequently accompanied by an elevated risk of thrombotic events. Matrix metalloproteinases (MMPs), a family of zinc-dependent endopeptidases, are implicated in numerous inflammatory and vascular pathophysiological processes. In this study, we analyzed the association between selected *MMP* polymorphisms, rs1799750, rs243865, rs3025058, rs3918242, and rs17576, and thrombotic risk as well as clinical characteristics in patients with MPNs. Genotyping was performed using the polymerase chain reaction-restriction fragment length polymorphism (PCR-RFLP) method. Among the polymorphisms analyzed, a statistically significant association was identified between the *MMP-9* rs3918242 CT genotype and an increased risk of arterial thrombosis (OR = 4.206, CI 1.337–13.234, *p* = 0.014). Moreover, rs3918242 CT was associated with thrombotic events (both arterial and venous thrombosis combined), suggesting a potential contributory role in the prothrombotic phenotype observed in MPNs (OR = 3.200, CI 1.110–9.258, *p* = 0.031). These findings indicate that genetic variation in *MMP-9*, particularly rs3918242, may serve as a predictive marker for vascular complications in MPN patients. Further studies with larger cohorts are warranted to confirm these associations and to elucidate the molecular mechanisms underlying the contribution of *MMP* polymorphisms to thrombosis in MPNs.

## 1. Introduction

According to the World Health Organization (WHO), classical Philadelphia chromosome-negative (*BCR-ABL1*-negative) myeloproliferative neoplasms (MPNs) are clonal myeloid stem cell disorders that include primary myelofibrosis (PMF), essential thrombocythemia (ET), and polycythemia vera (PV). MPNs are a group of hematologic malignancies characterized by the overproduction of red blood cells (RBCs), white blood cells (WBCs), platelets (PLTs), or a combination thereof due to the aberrant proliferation of myeloid progenitors. The *BCR-ABL1*-negative MPNs are known as rare disorders, with an estimated global incidence rate of 0.84, 1.03, and 0.47 per 100,000 population per year for PV, ET, and PMF, respectively [1,2,3]. The precise etiology of *BCR-ABL1*-negative MPNs is unknown; however, genetic predisposition, environmental exposures, and lifestyle factors have all been implicated in disease development. Each subtype exhibits distinct clinical and hematologic features, yet they share a unifying molecular hallmark: the somatic acquisition of a driver mutation. More than 90% of MPN patients harbor a mutation in genes encoding Janus kinase (JAK2), calreticulin (CALR), or the thrombopoietin receptor (MPL) [1,2,3]. While *JAK2* mutations are found across all three subtypes, *CALR* or *MPL* mutations are predominantly restricted to ET and PMF [4]. These mutant oncoproteins constitutively activate the JAK/STAT signaling pathway and alternative downstream cascades, leading to uncontrolled myeloproliferation and clinical manifestations, including an elevated risk of thrombosis and debilitating constitutional symptoms [5,6,7,8,9,10].

Matrix metalloproteinases (MMPs) are zinc- and calcium-dependent endopeptidases that degrade and remodel extracellular matrix (ECM) proteins [11,12]. In addition to ECM turnover, MMPs participate in numerous biological processes regulated by hormones, growth factors, and cytokines [13]. Their activity is tightly controlled by tissue-inhibitory metalloproteinases (TIMPs), which prevent excessive ECM degradation [14,15]. Based on substrate specificity and subcellular localization, MMPs are classified into four groups: collagenases (MMP-1, MMP-8, MMP-13, and MMP-18), gelatinases (MMP-2 and MMP-9), stromelysins (MMP-3, MMP-10, and MMP-11), and matrilysins (MMP-7 and MMP-26) [11,15,16]. Functionally, collagenases degrade fibrillar collagens essential for bone and ligament integrity [17]; gelatinases are involved in angiogenesis, neurogenesis, and cell death [18]; stromelysins degrade non-collagen ECM components [15,19]; and matrilysins process cell surface molecules and ECM components [20,21]. Apart from the MMPs’ role in physiological processes, such as embryogenesis, morphogenesis, angiogenesis, and wound repair, MMPs are implicated in pathological conditions, including inflammation, fibrosis, autoimmune diseases, and cancer [11,22,23]. Initially, MMPs were thought to facilitate metastasis by degrading ECM and basement membranes; however, they are now known to affect multiple aspects of tumor biology, including initiation, progression, angiogenesis, cytokine regulation, and the modulation tumor microenvironment [18,23,24]. Polymorphisms and the dysregulation of *MMP* gene expression have been associated with various cancers such as breast [25], colorectal [26], gastric [27], lung [28], and lymphoblastic leukemia [29]—as well as poor clinical outcomes [30,31]. However, the molecular mechanisms underlying *MMP*-related genetic variations and carcinogenesis remain incompletely understood and warrant further investigation.

Malignant hematological diseases, including MPNs, are often accompanied by genetic alterations such as somatic mutations, single nucleotide polymorphisms (SNPs), and inherited haplotypes [32]. Some of these variants are associated with thrombosis risk and disease progression [8]. Recent evidence has implicated MMPs in the pathogenesis of hematologic malignancies, including their roles in vascular remodeling and ECM degradation, both of which contribute to thrombosis [18,33,34,35]. MMP-2 and MMP-9, in particular, promote thrombus formation by modulating platelet aggregation and endothelial dysfunction [36,37,38,39,40,41,42,43,44]. Fan with colleagues [45] reported elevated MMP-9 expression in monocytes/macrophages of patients with ET, suggesting a novel mechanism of platelet production. MMPs also destabilize atherosclerotic plaques, contributing to cardiovascular and thrombotic risk [46,47]. Additionally, dysregulated MMP activity is implicated in venous thrombosis, such as deep vein thrombosis (DVT) and pulmonary embolism (PE) [46,47,48,49,50,51,52]. Despite these findings, few studies have directly analyzed the association between myeloproliferative neoplasms and changes in *MMP* genes [53,54]. Some evidence suggests that *MMP-9* variants are associated with increased MPN risk, bone marrow fibrosis, and thrombotic events. Furthermore, plasma MMP-9 levels correlate with platelet count and granulocyte mass in MPN patients, indicating its potential as a biomarker [54,55]. Using gene expression profiling, Skov et al. [56] identified the deregulated expression of *MMP-1*, *MMP-3*, *MMP-9*, as well as other ECM-related genes in MPN patients. This supports the hypothesis that abnormal ECM metabolism in MPN patients is driven by altered stromal gene regulation. In addition, Kelliher with coauthors [57] identified prothrombotic proteins—including MMP-1—in the MPN platelet proteome, and a recent study found that MMP-9 levels in ET patients correlated with *JAK2 p*.V617F allele burden and leukocyte count [58]. Given that platelet–leukocyte interactions contribute to thrombosis in MPNs [37], MMP-9 may influence thrombotic risk via leukocyte-mediated pathways. In conclusion, further research is needed to clarify the molecular mechanisms linking *MMP* gene alterations to thrombotic complications in MPNs.

Our study aims to analyze the association between the selected polymorphisms in *MMP-1*, *MMP-2*, *MMP-3*, and *MMP-9* genes (rs1799750, rs243865, rs3025058, rs3918242, and rs17576), thrombotic complications, and clinical characteristics in patients with MPNs. As a pilot study, our findings may contribute to a better understanding of MPN pathogenesis and assess whether *MMP* polymorphisms may be a potential biomarker for thrombosis risk.

## 2. Results

A total of 88 consented MPN patients were genotyped for selected *MMP* gene polymorphisms. The genotype distributions of *MMP-1* rs1799750, *MMP-2* rs243865, *MMP-3* rs3025058, *MMP-9* rs3918242, and rs17576 polymorphisms are presented in Table 1. The observed genotype frequencies in the MPN group were comparable to those reported in the European population according to the 1000 Genomes Project Database. All polymorphisms were in Hardy–Weinberg equilibrium (HWE).

Further, we investigated the association between selected *MMP* gene polymorphisms and clinicopathological features in patients with MPNs, as detailed in Supplementary Table S1. The findings suggest potential genotype–phenotype correlations that may implicate *MMP* variants in modulating disease phenotype and thrombotic risk. Specifically, the *MMP-1* rs1799750 and *MMP-2* rs243865 polymorphisms were significantly associated with an increased risk of venous thrombosis (*p* = 0.041 and *p* = 0.022, respectively). Additionally, the *MMP-9* rs3918242 variant showed a significant association with both arterial thrombosis and overall thrombotic events (arterial and venous combined; *p* < 0.05). Notably, the *MMP-9* rs17576 polymorphism was significantly correlated with platelet count (*p* < 0.05), suggesting a possible role in megakaryopoiesis or platelet turnover. While mean platelet volume (MPV) is recognized as an indirect marker of platelet reactivity—where larger platelets exhibit greater prothrombotic potential, an increased secretion of vasoactive mediators, and a higher expression of adhesion molecules [59,60,61,62,63]—no significant associations were observed between MPV and any of the investigated *MMP* polymorphisms. These preliminary findings support a potential role for *MMP* genetic variants in the thrombotic profile and hematologic parameters of MPNs, warranting further analysis.

Univariate logistic regression analysis revealed the association between *MMP* polymorphisms and thrombotic risk in MPN patients. Specifically, the *MMP-9* rs3918242 CT genotype (compared to CC) was significantly associated with an increased risk of arterial thrombosis (OR = 4.206, CI 1.337–13.234, *p* = 0.014) and overall thrombotic events (both arterial and venous thromboses combined) (OR = 3.200, CI 1.110–9.258, *p* = 0.031). Moreover, MPN patients carrying the *MMP-1* rs19799750 1G2G genotype (vs 2G2G) tended to have an increased risk of arterial thrombosis (*p* = 0.059), while the *MMP-3* rs3025058 6A6A genotype (compared to 5A5A) showed a tendency towards decreased arterial thrombosis (*p* = 0.058) (Table 2). One limitation of our study is the relatively small patient group, which may have limited statistical power. Notably, near-significant *p*-values may become significant with larger sample sizes. We must emphasize that there were no statistically significant differences between patients with and without thrombosis in terms of age at diagnosis, *JAK2* p.V617F mutational status, or WBC count (*p* > 0.05 for all) (Table 3), so we excluded these confounding factors from binary logistic regression analysis applied for odds ratio evaluation. Although the mean corpuscular volume (MCV) and mean corpuscular hemoglobin (MCH) were significantly different between the two groups (*p* = 0.016 and *p* = 0.013, respectively) (Table 3), it was not included in the binary logistic regression analysis, as MCV and MCH are not considered to have a direct association with thrombotic risk or platelet count in MPNs.

## 3. Discussion

Several studies have recently analyzed the role of MMPs in various biological processes and their association with cancer. This raises the question of whether MMPs are involved in the pathogenesis of MPNs and whether they could serve as potential biomarkers for disease monitoring. Nevertheless, the role of MMPs in MPNs remains largely unexplored, and final conclusions have not been presented. In this study, we aimed to deepen the analysis of genetic alterations in genes encoding several MMPs and analyze their association with *BCR-ABL1*-negative MPNs.

Among MMPs, gelatinases MMP-2 and MMP-9 are the most extensively studied in various pathological conditions, such as diabetes, cardiovascular diseases, gliomas, and multiple cancers [25,64,65,66,67]. Recent research has suggested a significant role of MMPs in the development of hematologic malignancies, with a primary focus on leukemia [29,33]. However, there are still limited data regarding the relationship between *MMP* gene polymorphisms and hematological diseases. It has been assumed that MMPs and their TIMPs, modulated by SNPs, are critical in leukemia development [68]. Chaudhary et al. [68] conducted a comprehensive study investigating mRNA expression and secretion levels of MMP-2 and MMP-9 in bone marrow mononuclear cells, as well as genotypic associations of *MMP-2* rs243865 and *MMP-9* rs3918242 variants in myelodysplastic syndrome (MDS) and acute myeloid leukemia (AML) during the disease progression. Their findings are particularly relevant because MDS/MPN overlap syndromes—especially those with *JAK2* mutations—are associated with an increased risk of thrombosis. One of the aims of our study was to examine whether *MMP* polymorphisms influence thrombotic risk in MPN patients. Chaudhary with coauthors [68] reported an association between the *MMP-9* rs3918242 variant and both environmental factors and addiction habits, suggesting a possible role in the initiation of the progression of hematological malignancies. They also found that the *MMP-2* rs243865 T allele was significantly associated with AML compared to healthy controls. Other studies have also identified *MMP-2* rs243865 as a potential molecular risk factor for B-cell non-Hodgkin’s lymphoma and T-cell acute lymphoblastic leukemia [29,69]. These findings suggest that this SNP could be implicated not only in hematological disease onset but also in disease progression. Furthermore, Chaudhary et al. [68] presented that MMP-2 and MMP-9 levels increased as the disease progressed, indicating their potential as biomarkers for assessing leukemic burden in AML. These enzymes may also affect hematopoietic cell behavior, a hypothesis supported by Travaglino et al. [70], who concluded that deregulated *MMP* expression in MDS could serve as an early predictor of hematopoietic dysfunction and a valuable diagnostic and prognostic marker, as well as a therapeutic target. Both groups of researchers emphasized the need for larger patient cohorts to confirm associations between *MMP* polymorphisms and clinical features of hematologic diseases. Unfortunately, the involvement of *MMP-2* and *MMP-9* polymorphisms in the pathogenesis and prognosis of MPN remains poorly understood. One study by Sag et al. [53] analyzed the *MMP-9* rs3918242 variant in MPN patients and found that the CC genotype showed borderline significance in PV patients versus the control group. Although approximately half of PV and ET patients experience arterial or venous thrombosis, leading to a high proportion of MPN-related deaths [71], Sag with coauthors did not find a significant association between *MMP-9* rs3918242 and thrombotic events. Other studies also reported no clear relationship [53,72]. Nevertheless, independent research has identified *MMPs* as contributors to thrombotic and vascular events, regardless of the underlying disease. For instance, Zhang et al. [73] reported that the T allele of the *MMP-9* rs3918242 polymorphism is associated with an increased risk of severe cardiac atherosclerosis. Moreover, Wang et al. [74] showed a higher risk of three-vessel disease in T allele carriers. Malaponte and coauthors [75] determined that the CC genotype of *MMP-9* rs3918242 was linked to deep vein thrombosis in cancer patients. Additionally, interactions between rs3918242 and rs3787268 in the *MMP-9* gene were associated with hemorrhagic transformation in acute ischemic stroke patients with atherothrombosis, small artery disease, and cardioembolic stroke [76]. Moreover, Tsuei and colleagues [77] determined that the *MMP-2* rs243865 polymorphism, but not the *MMP-9* rs17576, was a risk factor for sinus thrombosis in dural arteriovenous fistula patients. Multiple studies have confirmed the role of *MMP-1* and *MMP-2*, along with inflammatory factors, in the pathogenesis of deep vein thrombosis [50]. Measuring their levels in the peripheral blood has clinical relevance for the diagnosis and prognosis. Furthermore, Halucha et al. [78] showed that MMP-2 inhibition reduces platelet activation in ischemia/reoxygenation conditions, and animal models have demonstrated that resistin promotes thrombosis by upregulating *MMP-2*, *MMP-9*, and *PAI-1* expression [79]. Yu et al. [51] further confirmed the involvement of MMP-2, MMP-9, and TIMPs in venous wall remodeling. Maral et al. [54] explored associations between *MMP* polymorphisms and chronic myeloproliferative diseases and *MMP-2* rs2285053, *MMP-9* rs17576, and ET, PV, and secondary polycythemia. They also proposed that *MMP* variants may influence bone marrow fibrosis and thrombosis risk. Years earlier, Sawicki with colleagues [55] stated that there is a significant correlation between plasma MMP-9 concentration and platelet count, indicating that MMP-9 may reflect platelet mass and granulocyte levels. Stromal cell-derived factor 1 (SDF-1) has been shown to regulate megakaryocyte migration and MMP-9 expression, leading to increased platelet production [42,80], which is associated with thrombopoiesis. Our study, which focused on polymorphisms in the genes coding gelatinases MMP-2 and MMP-9, found that the *MMP-9* rs3918242 CT genotype was significantly associated with an increased risk of arterial thrombosis (OR = 4.206, CI 1.337–13.234, *p* = 0.014) and thrombotic events (both arterial and venous thromboses combined) (OR = 3.200, CI 1.110–9.258, *p* = 0.031, respectively). To analyze a possible link with platelet activation [54,55], we explored the association between selected *MMP* polymorphisms and platelet count or MPV but found no significant correlations. Taken together, while comparing studies is challenging because of differences in methodologies and patient populations, our data support the hypothesis that the *MMP-9* rs3918242 polymorphism may be related to thrombotic events in MPNs. Regarding the molecular biology of this genetic variant, rs3918242 is known to enhance *MMP-9* gene expression, which may contribute to endothelial dysfunction, inflammation, and platelet activation—key processes in MPN-related thrombogenesis [11,18,22,23,36,37,38,39]. Increased *MMP-9* activity in rs3918242 CT genotype carriers may lead to endothelial dysfunction, facilitating a procoagulant vascular surface, increased inflammatory cytokine activity, and enhanced thrombotic potential. Thus, it can be assumed that the *MMP-9* rs3918242 CT genotype may serve as a genetic modifier, increasing the risk of thrombosis via MMP-9-mediated pathways. Although the *MMP-9* rs3918242 polymorphism has not been extensively studied, our findings suggest that this genetic variant warrants further investigation due to its potential relevance in the pathogenesis of *BCR-ABL1*-negative MPNs. A deeper understanding of gelatinase expression and related genetic alterations may open avenues for novel diagnostic and therapeutic strategies.

Regarding *MMP-1*, the rs1799750 variant is located in the promoter region of the gene, coding tissue collagenase I. The mentioned polymorphism has been analyzed in the context of cancer and other diseases [81,82,83,84,85,86]. While some studies suggest potential associations, particularly with breast cancer [81] and knee osteoarthritis [84], the overall evidence remains inconsistent. A possible protective effect of the *MMP-1* rs1799750 1G allele has been suggested in childhood acute lymphoblastic leukemia [87]. A comprehensive pan-cancer analysis revealed that *MMP-1* is significantly upregulated in various cancers, indicating its potential involvement in tumor progression [88]. Moreover, Zhang et al. [50] analyzed serum levels of MMP-1 and MMP-2 in patients with lower extremity deep vein thrombosis. The data showed that in DVT patients, compared to healthy controls, serum levels of MMP-1 and MMP-2 were significantly higher. After treatment, these levels decreased notably. This suggests that MMP-1 and MMP-2 may play an important role in the development of DVT. Austin with colleagues [89] found that MMP-1 activates protease-activated receptor 1 (PAR1), which may contribute to thrombus formation. However, no studies have directly investigated *MMP-1* expression in MPNs, and data on the *MMP-1* rs1799750 variant association with thrombosis is scarce. While some variants affect MMP-1 plasma levels [90,91], a direct link to thrombosis risk remains unclear. To the best of our knowledge, no more research has been carried out on *MMP-1* rs1799750 and the pathogenesis or progression of MPNs. In our study, the *MMP-1* rs1799750 1G2G genotype (compared to 2G2G) showed a trend toward increased arterial thrombosis risk (OR = 3.200, 95% CI 0.956–10.714, *p* = 0.059), indicating a possible association that warrants further investigation in larger cohorts. Currently, there is a lack of specific studies examining MMP-1 in MPNs, which complicates direct comparisons with our findings. Therefore, further research with larger and more diverse populations is necessary to elucidate the associations between MMP-1 and MPNs and to better understand the underlying molecular mechanisms.

With wide substrate specificity, MMP-3 (stromelysin I) is a crucial member of the MMP family. Most importantly, MMP-3 is related to the activation of MMP-1 and is capable of degrading proteoglycan, fibronectin, laminin, and type IV collagen [86]. The *MMP-3* rs3025058 promoter polymorphism has been investigated in several diseases, including certain cancers, cardiovascular conditions, musculoskeletal injuries, and other diseases [86,92,93,94,95,96]. However, there is no evidence linking the rs3025058 polymorphism specifically with MPNs. Also, specific studies directly examining *MMP-3* expression in MPNs are limited. A comprehensive pan-cancer analysis of *MMP* gene expression profiles across various neoplasms revealed that *MMP-3* is significantly up-regulated in at least 10 cancer types; however, it did not identify any notable findings related to MMP-3 in MPNs [88]. As mentioned before, the *MMP-3* rs3025058 has been studied for its association with various cardiovascular conditions, including thrombosis. Zee et al. [97] reported a significant association between this polymorphism and recurrent venous thromboembolism (VTE). In a multi-locus genetic study investigating 86 genetic variants across 56 genes, *MMP-3* rs3025058 was identified as one of four variants linked to an increased risk of recurrent VTE. Another important case-control study was carried out by Li et al. [98]. It was determined that the 6A allele of *MMP-3* rs3025058 may be associated with an increased risk of DVT, and the MMP-3 serum level in DVT patients was markedly higher than that in the control group. These findings suggest that *MMP-3* expression may influence the susceptibility to and resolution of thrombotic events; however, the authors emphasize that further research is required to elucidate the underlying mechanisms and clarify the clinical significance of *MMP-3* expression in thrombosis. In contrast to the studies mentioned, our results may support the assumption that the *MMP-3* rs3025058 6A6A genotype (vs. 5A5A) tends to decrease the risk of arterial thrombosis (OR = 0.400, 95% CI 0.155–1.031, *p* = 0.058). A statistically significant association between lower-risk arterial thrombosis and rs3025058 6A6A would be expected with a higher number of MPN patients. It is important to note that the patient cohorts and types of thrombosis tested differ between our study and Zee et al.’s [97]. In our study, no association was found between *MMP-3* rs3025058 and venous thrombosis. Additionally, while one meta-analysis of case-control studies suggested a potential association between *MMP-3* polymorphisms, including rs3025058, and ischemic stroke [99], another meta-analysis found no such link [100]. These conflicting findings highlight the inconclusive nature of current evidence regarding the relationship between *MMP-3* rs3025058 and cardiovascular events. Nevertheless, the mentioned polymorphism may influence susceptibility to thrombotic conditions such as recurrent VTE and ischemic stroke. Further research is needed to elucidate the potential involvement of *MMP-3*, particularly rs3025058, in the pathophysiology of MPNs.

However, our study has limitations that need to be acknowledged. Although this study supports an association between the *MMP-9* rs3918242 and thrombotic events, the limited number of patients with *BCR-ABL1*-negative MPN leads to the assumption that our findings should be taken as hypothesis-generating rather than conclusive. Moreover, the study population may not fully represent the broader MPN patient population because patients were recruited from a single center. Considering that *BCR-ABL1*-negative MPNs are classified as rare diseases, a larger research design would necessitate multicentric recruiting. Another limitation is the lack of a healthy control group, which restricts the ability to compare genotype frequencies and thrombosis risk against a baseline population. The inclusion of an appropriate control group in future studies would enhance the interpretation of the genetic association with the risk of MPNs and MPN-related complications. Moreover, in the absence of a healthy individuals and a disease-control group, it is not possible to determine whether the presence of the studied *MMP* polymorphisms or associated hematologic features (such as elevated platelet counts) is specific to MPN. But at the same time, we want to underline that our study was designed to evaluate the association of selected genetic polymorphisms with thrombotic risk and clinical characteristics within a cohort of patients already diagnosed with MPN, rather than establishing disease-specific markers. Furthermore, the absence of a functional analysis, such as gene expression or protein-level analysis, limits our understanding of the mechanistic relevance of the *MMP-9* variant in thrombotic predisposition. Additional functional studies are warranted to better elucidate the biological significance of these findings.

Despite the limitations mentioned, our study significantly contributes to the clarification of the associations between *MMP* gene polymorphisms and MPN. This study identified *MMP* gene variants that have not been previously associated (or tended to be associated) with MPN progression, especially with the risk of thrombotic events. Furthermore, our findings provide a basis for developing novel hypotheses regarding the mechanisms behind these diseases, which may be explored in larger, more comprehensive studies. Our data may assist in identifying potential prognostication biomarkers or therapeutic targets, especially in terms of how *MMP* gene variants impact MPN complications. Also, by including a cohort of 88 patients, the study creates a valuable dataset that can be used in future meta-analyses. Therefore, even as a pilot study, this research provides important insights into the association between *MMP* variants and MPN pathogenesis. It significantly contributes to the direction of future research and the potential development of new diagnostic and therapeutic approaches.

## 4. Materials and Methods

### 4.1. Study Population

The present study was a retrospective analysis of 88 patients (patient data were collected over 14 years) with PMF, ET, or PV diagnoses confirmed according to the WHO 2016 diagnostic criteria at the Department of Hematology of the Institute of Oncology, the Lithuanian University of Health Sciences, Kaunas, Lithuania. Detailed medical information, including age at diagnosis, sex, smoking status, the history of arterial and venous thrombosis, MPV, MCV, MCH, hemoglobin (Hb), hematocrit (Ht), RBC, WBC, monocyte, basophil, and PLT count, as well as *JAK2* p.V617F mutational status, was collected from medical records. Of a total of 88 patients, 7 (8.0%) patients had PMF, 45 (51.1%) patients had ET, and 36 (40.9%) were PV patients. Almost half of the MPN patients had thrombotic complications, i.e., 10 with venous (11.36%) and 29 with arterial (32.95%) thrombosis. The clinical characteristics mentioned above are presented in Table 3.

This pilot study was approved by the Kaunas Regional Ethics Committee for Biomedical Research (protocol number BE-2-9, date: 6 March 2013) and conducted following good clinical and laboratory practices and the principles of the Declaration of Helsinki. Signed informed consent forms for participation in this study were obtained from all patients.

### 4.2. Single Nucleotide Polymorphisms and Genotyping

*MMP-1* rs1799750, *MMP-2* rs243865, *MMP-3* rs3025058, *MMP-9* rs3918242, and rs17576 were identified through the dbSNP database (https://www.ncbi.nlm.nih.gov/snp/ (accessed on 7 April 2025)). Polymorphisms were selected based on a minor allele frequency (MAF) ≥2%. Previous studies showing an association of these polymorphisms with cancer were also considered [53,54,101].

The genotyping of the mentioned polymorphisms was performed at the Oncology Research Laboratory of the Oncology Institute at the Lithuanian University of Health Sciences. Venous blood samples were collected in vacutainers with EDTA as an anticoagulant and stored at −20 °C until further processing. Genomic DNA was extracted from the peripheral blood leukocytes of each subject using a commercially available DNA extraction kit (Thermo Fisher Scientific, Waltham, MA, USA). Extracted DNA was aliquoted and stored at −20 °C as a working stock. The genotyping of *MMP-1*, *MMP-2*, *MMP-3*, and *MMP-9* single nucleotide polymorphisms was performed via the typical polymerase chain reaction restriction fragment length polymorphism (PCR-RFLP) method. Detailed data on genotyping, primer sequences, restriction enzymes (Thermo Fisher Scientific, Waltham, MA, USA), and length of PCR-RFLP products are shown in Table 4 [54,102,103,104,105]. The digested PCR products were analyzed by electrophoresis on a 3% agarose (Thermo Fisher Scientific, Waltham, MA, USA) gel and visualized under UV light after staining with ethidium bromide (Sigma-Aldrich, St. Louis, MO, USA).

### 4.3. Statistical Analysis

The Statistical Package for Social Sciences (IBM SPSS Statistics) version 29.0.0.0 was used for the association analyses. A Hardy–Weinberg equilibrium (HWE) for each polymorphism in the tested group was assessed. The chi-square (χ^2^) test was used to evaluate the statistical significance. The Fisher exact test was used in cases where the scattering exceeded 25%. Binary logistic regression analyses were conducted to calculate the odds ratio linking various *MMP* genotypes with laboratory variables and thrombosis. A *p*-value below 0.05 was considered to have statistical significance.

## 5. Conclusions

This study revealed findings indicating that the *MMP-9* rs3918242 variant may be a promising biomarker for the prognosis of *BCR-ABL1*-negative MPNs, especially in assessing thrombotic risk. It is also important to mention that *MMP-1* rs1799750 created a tendency towards an increased risk of arterial thrombosis in MPN patients. On the contrary, the *MMP-3* rs3025058 showed a tendency for decreased arterial thrombosis. Given the pilot nature of this study, future studies with larger MPN patient groups will certainly provide more accurate information for establishing an association between *MMP* polymorphisms and MPNs.

## Figures and Tables

**Table 1:** Genotype distribution of *MMP-1* rs1799750, *MMP-2* rs243865, *MMP-3* rs3025058, *MMP-9* rs3918242, and rs17576 polymorphisms among MPN patients.

Type of MMP	Polymorphism	Genotype	Frequency, (%)	HWE *p*-Value
Collagenase	*MMP-1* rs1799750	1G1G	26 (29.6)	χ^2^ = 3.123 *p* = 0.08
		1G2G	37 (42.0)	
		2G2G	25 (28.4)	
Gelatinase	*MMP-2* rs243865	CC	55 (62.5)	χ^2^ = 1.077 *p* = 0.299
		CT	27 (30.7)	
		TT	6 (6.8)	
Stromelysin	*MMP-3* rs3025058	5A5A	20 (22.7)	χ^2^ = 0.0004 *p* = 0.985
		5A6A	44 (50.0)	
		6A6A	24 (27.3)	
Gelatinase	*MMP-9* rs3918242	CC	66 (75.0)	χ^2^ = 1.147 *p* = 0.284
		CT	19 (21.6)	
		TT	3 (3.4)	
	*MMP-9* rs17576	AA	37 (42.0)	χ^2^ = 0.395 *p* = 0.530
		AG	38 (43.2)	
		GG	13 (14.8)	

Note: HWE—Hardy–Weinberg equilibrium.

**Table 2:** Univariate logistic regression analysis. The odds ratio for the association between polymorphisms and MPN characteristics. Only statistically significant associations and tendencies are shown.

Characteristic	Polymorphism	Genotype	OR	95% CI	*p*-Value
Arterial thrombosis	*MMP-1* rs1799750	2G2G	1.000	Reference	-
		1G2G	3.200	0.956–10.714	0.059
Arterial thrombosis	*MMP-3* rs3025058	5A5A	1.000	Reference	-
		6A6A	0.400	0.155–1.031	0.058
Arterial thrombosis	*MMP-9* rs3918242	CC	1.000	Reference	-
		CT	4.206	1.337–13.234	**0.014**
Thrombotic events (both arterial and venous thromboses combined)	*MMP-9* rs3918242	CC	1.000	Reference	-
		CT	3.200	1.110–9.258	**0.031**

Note: OR—odds ratio, CI—confidence interval. The bold values represent statistically significant results (*p* < 0.05).

**Table 3:** Clinical characteristics.

Characteristics	Patients with Thrombosis (*n* = 36)	Patients without Thrombosis (*n* = 52)	*p* Value
Median age in years (min–max)	73 (35–87)	63 (27–86)	0.684 ^a^
Males: *n* (%)	18 (48.6)	19 (51.4)	0.208 ^a^
Females: *n* (%)	18 (35.3)	33 (64.7)	
Hb (g/dL): mean (SD)	149.09 (36.98)	148.17 (36.16)	0.908 ^b^
Ht (%): mean (SD)	50.17 (30.42)	47.76 (12.19)	0.625 ^b^
RBC count (10^12^/L): median (min–max)	5.37 (10.47)	5.17 (1.23)	0.625 ^c^
MCV (fL): median (min–max)	82 (8.43)	86 (18.56)	**0.016 ^c^**
MCH (pg): median (min–max)	27 (3.89)	28 (9.33)	**0.013 ^c^**
MPV (fL): median (min–max)	8.70 (7.10–12.50)	9.70 (0.12–11.20)	0.633 ^c^
PLT count (10^9^/L): median (min–max)	549 (327.64)	581 (304.91)	0.694 ^c^
WBC count (10^9^/L): mean (SD)	10.89 (3.54)	10.61 (5.26)	0.791 ^b^
Monocyte count (10^9^/L): median (min–max)	0.59 (0.31)	0.58 (0.69)	0.593 ^c^
Basophils (10^9^/L): median (min–max)	0.07 (0.38)	0.10 (0.39)	0.272 ^c^
Smokers: *n* (%)	2 (50.0)	2 (50.0)	0.695 ^a^
*JAK2* p.V617F-positive: *n* (%)	27 (51.9)	25 (48.1)	0.061 ^a^

Note: ^a^—ζ^2^ Test for independence (homogeneity) of two features, ^b^—*t*-Test for two independent samples, ^c^—Non-parametric Mann–Whitney U test. The bold values represent statistically significant results (*p* < 0.05).

**Table 4:** Primer sequences, restriction enzymes used for genotyping, and length of PCR-RFLP products.

Substitution	Primer Sequence	Restriction Enzyme	Fragment Size
*MMP-1* rs1799750	F: 5′-TGACTTTTAAAACATAGTCTATGTTCA-3′ R: 5′-TCTTGGATTGATTTGAGATAAGTCATAGC-3′	*Alu*I	1G1G-241, 28 bp
			1G2G-270, 241, 28 bp
			2G2G-270 bp
*MMP-2* rs243865	F: 5′-ATATTCCCCACCCAGCAGTC-3′ R: 5′-TTGGGAACGCCTGACTTCAG-3′	*Acc*I	CC-122 bp
			CT-122, 103, 19 bp
			TT-103, 19 bp
*MMP-3* rs3025058	F: 5′-GGTTCTCCATTCCTTTGATGGGGGGAAAGA-3′ R: 5′- CTTCCTGGAATTCACATCACTGCCACCACT-3′	*Psy*I	5A5A-97, 32 bp
			5A6A-129, 97, 32 bp
			6A6A-129 bp
*MMP-9* rs3918242	F: 5′-GCCTGGCACATAGTAGGCCC-3′ R: 5′-CTTCCTAGCCAGCCGGCATC-3′	*Sph*I	CC-436 bp
			CT-436, 242, 194 bp
			TT-242, 194 bp
*MMP-9* rs17576	F: 5′- AGACCATCCATGGGTCAAAG-3′ R: 5′- GATTGGCCTTGGAAGATGAA-3′	*Sma*I	AA-105, 58 bp
			AG-168, 105, 58 bp
			GG-168 bp

Note: F—forward, R—reverse, bp—base pair.

## Data Availability

The data are contained in the article or Supplementary Material. Further inquiries can be directed to the corresponding author.

## Supplementary Materials

The supporting information can be downloaded at: https://www.mdpi.com/article/10.3390/ijms26146646/s1.
